# Phase-matched third-harmonic generation in silicon nitride waveguides

**DOI:** 10.1515/nanoph-2024-0120

**Published:** 2024-05-31

**Authors:** Surendar Vijayakumar, Kaustubh Vyas, Daniel H. G. Espinosa, Orad Reshef, Meiting Song, Kashif Masud Awan, Saumya Choudhary, Jaime Cardenas, Robert W. Boyd, Ksenia Dolgaleva

**Affiliations:** 248498 Institute of Optics, University of Rochester, 480 Intercampus Dr, Rochester, NY 14627, USA; Department of Electrical and Computer Engineering, 8786 University of California Santa Barbara, Santa Barbara, CA, USA; 7548 Institute of Materials Science and Engineering, Washington University, St Louis, MO 63130, USA; Department of Physics, 67125 University of Ottawa, 25 Templeton Street, K1N 6N5, Ottawa, ON, Canada; School of Electrical Engineering and Computer Science, 67125 University of Ottawa, 800 King Edward Ave., Ottawa, ON, K1N 6N5, Canada

**Keywords:** harmonic generation, waveguide, frequency conversion, silicon nitride, nonlinear optics

## Abstract

Third-harmonic generation (THG) in silicon nitride waveguides is an ideal source of coherent visible light, suited for ultrafast pulse characterization, telecom signal monitoring and self-referenced comb generation due to its relatively large nonlinear susceptibility and CMOS compatibility. We demonstrate third-harmonic generation in silicon nitride waveguides where a fundamental transverse mode at 1,596 nm is phase-matched to a TM_02_ mode at 532 nm, confirmed by the far-field image. We experimentally measure the waveguide width-dependent phase-matched wavelength with a peak-power-normalized conversion efficiency of 5.78 × 10^−7^ %/W^2^ over a 660-μm-long interaction length.

## 1 Introduction

Nonlinear frequency conversion of commercially available lasers provides access to wavelengths not readily achievable in gain media. Second-harmonic generation (SHG) occurs in non-centrosymmetric materials, while third-harmonic generation (THG) occurs in all materials irrespective of symmetry constraints [1]. THG finds applications in high-resolution microscopy, providing high-contrast three-dimensional images [2]. Ultrafast pulses can be characterized using their third harmonic as an autocorrelation signal [3], and the THG of telecom signals can be monitored using efficient visible detectors at rates up to 640 GHz/s [4]. Self-referenced frequency combs require phase-locked harmonic signals generated on-chip for stabilization of next-generation atomic clocks that are resonant to visible radiation [5]. THG in bulk materials is often implemented by SHG of the pump beam, followed by a sum-frequency generation (SFG) of the second harmonic with the unconverted pump [6]. Such cascaded processes require multiple crystals and optical components to focus and align the beam in each crystal, limiting its applications due to spatiotemporal walk-off effects, mode-mismatch, and misalignment. Implementing THG in single step is desirable, ensuring stability and mode-matching to the application. Single-step THG requires (1) materials transparent to both the fundamental and third-harmonic wavelengths; (2) long phase-matched interaction length; (3) large third-order nonlinear susceptibility; and (4) low mode volume to increase the peak intensity of the pump mode. These conditions are satisfied in nanophotonic components [7].

Si-based nanophotonic devices are attractive due to their large mode confinement and high nonlinear susceptibility up to 9.0 × 10^−18^ m^2^/W [8]. THG has been reported in Si metasurfaces with efficiency on the order of 10^−7^ at large peak intensities (2–5.5 GW/cm^2^) [9–14]. High-Q metasurfaces cannot convert the whole spectrum of pulsed lasers as their bandwidths are limited to the resonance linewidth [15], and the light cannot be focused to arbitrary intensities over interaction lengths longer than the Rayleigh distance [16]. These shortcomings are overcome by waveguides that support large intensities for long propagation lengths. Si photonic crystal slow-light waveguides [17] have enabled the THG of picosecond pulses with a conversion efficiency of 10^−7^. Nanophotonic Si waveguides [18] have demonstrated a large third-harmonic conversion efficiency (2.8 × 10^−7^), supporting peak intensities up to 109.13 GW/cm^2^ over a length of 200 μm. However, Si absorbs visible light and suffers from two-photon-induced free-carrier absorption in the infrared (IR) range. This limits the nonlinear interaction length to a few microns and saturates the conversion efficiency at large excitation powers [13].

THG has been extensively studied in optical fibers, despite the low nonlinear coefficient of silica (2.7 × 10^−20^ m^2^/W) [19], through phase-matching of an IR fundamental mode and its third harmonic as a high-order mode (HOM). Optical fibers are transparent in the visible wavelength range, experiencing no two-photon absorption (TPA) at IR wavelengths, and can support a long interaction length. However, such a phase-matching mechanism requires a large core-cladding refractive index difference, which is not present in standard SMF-28 fibers [20]. Microstructured optical fibres (MOF), with sub-wavelength air holes, are used to generate third harmonics due to their large mode confinement and high refractive index contrast [21–25]. THG has also been observed in fibers composed of highly nonlinear materials such as Ge-doped silica fibers [26], [27], telluride fibers [28], [29], and chalcogenide fibers [30], [31], as well as tapered silica fibers [32], [33] and hollow-core fibers filled with noble gases [34]. Fibers with uniform core radius are only reported up to a few tens of centimeters [35], causing phase-mismatch at the long interaction lengths required to compensate for low nonlinearity and large mode area.

Integrated wide-bandgap dielectric nanostructures are transparent to wavelengths above 350 nm, exhibiting moderate nonlinearity between 2–4 × 10^−19^ m^2^/W [36]–[38], and can be fabricated in CMOS foundries. THG in waveguides can occur over a long interaction length, aided by modal phase-matching [39], and the phase-matched wavelength can be tailored by changing the waveguide width. THG was demonstrated in resonant silicon nitride antennas for UV generation [40], silicon nitride ring resonators [41], and Si_3_N_4_/AlN bilayer ring resonators with a large efficiency of 180 %/W^2^ [42]. THG of pulses has been demonstrated in TiO_2_ waveguides [43], where the phase-matched wavelength was controlled by the waveguide width over a limited interaction length of 60 μm. Cascaded THG has also been implemented in periodically poled lithium niobate (PPLN) waveguides [44]. Other implementations, such as Si-based waveguides [18], engender limited interaction length due to absorption, and KTP birefringently phase-matched waveguides [45] are inefficient due to low nonlinearity and large mode volume.

In this manuscript, we report on the design and realization of silicon nitride waveguides tailored for phase-matched THG and characterize the waveguides using an ultrafast laser. We observe the far field of the generated third harmonic to confirm phase-matching to the desired mode per the design. Further, we demonstrate waveguide-width-dependent phase-matched THG wavelength by characterizing the waveguides of different widths and investigating the tunability of the generated wavelength.

## 2 Device principles

In degenerate THG, the pump light of wavelength *λ*_p_ is upconverted into the signal, which is one-third of the pump wavelength (*λ*_p_/3) [1]. This process requires energy and momentum conservation, where the phases of both the pump and signal modes must be matched as they propagate along the waveguide. Phase-matching requires that the effective indices of both the fundamental and third-harmonic modes are equal. The length over which the fundamental and third-harmonic modes accumulate a phase mismatch is defined as the coherence length [1]

(1) $$L_{\text{coh}}=\frac{\pi }{\Delta k},$$

where Δ*k* is the effective wavevector mismatch. Normal chromatic dispersion leads to a decrease in the material’s refractive index with an increase in wavelength [23]. Additionally, the electromagnetic field at shorter wavelengths experiences stronger confinement in the waveguide, while longer wavelengths are weakly guided, leading to strong geometric dispersion. To compensate for the geometric and chromatic dispersions, we need a mechanism to match the effective refractive index (*n*_eff_) at both the pump and signal wavelengths [20]. Coherence length is commonly increased by dispersion compensation through birefringence or by modulating the nonlinear index by periodic poling, neither of which is possible in silicon nitride [46]. Silicon nitride is amorphous and domain inversion is not possible in third-order nonlinearities. In multimode waveguides, matching the effective refractive indices of a high-order mode (HOM) in the visible and the fundamental mode in the IR wavelength ranges is possible. As an HOM approaches the long-wavelength cutoff, its effective refractive index decreases more rapidly than that of the fundamental mode due to an increased evanescent field component of the former. This allows phase-matching of the two modes at a waveguide width near the long-wavelength cutoff of the HOM [23]. The efficiency of the phase-matched THG depends on the mode-field overlap between the pump and signal mode’s transverse electric field profiles, defined by the nonlinear overlap coefficient [47]

(2) $$\eta_{\mathrm{ijkl}}=\frac{\iint dxdyE_{s}^{i}E_{p}^{j^{*}}E_{p}^{k^{*}}E_{p}^{l^{*}}}{{\left[\iint dxdy|E_{s}{|}^{2}\right]}^{1/2}{\left[\iint dxdy|E_{s}{|}^{2}\right]}^{3/2}},$$

where $E_{p}^{i}$ and $E_{s}^{j}$ represent transverse electric field polarization components of the pump and signal fields, taken along different crystalline axes, labelled by the superscripts *i*, *j*, *k*, and *l*. The inverse of this coefficient is traditionally called the effective mode area (*A*_eff_ = 1/*η*_*ijkl*_) in waveguide optics. To ensure a non-zero field overlap, the transverse mode profile of the HOM must be symmetric with respect to the vertical and horizontal axes of the waveguide cross-section. The power of the THG signal mode generated in a phase-matched waveguide is given by [48]

(3) $$P_{\text{s}}=\frac{\gamma^{2}L^{2}}{9}P_{\text{p}}^{3},$$

where *L* is the length of the waveguide, *P*_s_ and *P*_p_ are the power of the signal and pump, respectively. The effective nonlinearity of the waveguide is given by

(4) $$\gamma =\frac{2\pi n_{2}}{\lambda_{\text{s}}}\eta_{\mathrm{ijkl}},$$

where we use the nonlinear index of stoichiometric silicon nitride *n*_2_ = 2.4 × 10^−19^ m^2^/W [37].

As a consequence of modal phase-matching, the third-harmonic is generated in the TM_02_ mode, which needs to be converted to a TM_00_ mode to interface with other applications. Mode conversion is implemented in a directional coupler [49], by evanescently coupling the TM_02_ mode with a TM_00_ mode in an adjacent waveguide. The effective refractive index of the coupled modes needs to be matched for strong coupling, conditioned on which the coupled modes form even and odd supermodes. 100 % power conversion occurs at a length given by

(5) $$L_{100\;\text{\%}}=\frac{\lambda_{\text{s}}}{2\Delta n_{\text{eff}}},$$

where Δ*n*_eff_ is the effective refractive index between the two supermodes.

## 3 Design and fabrication

**Figure 1:**
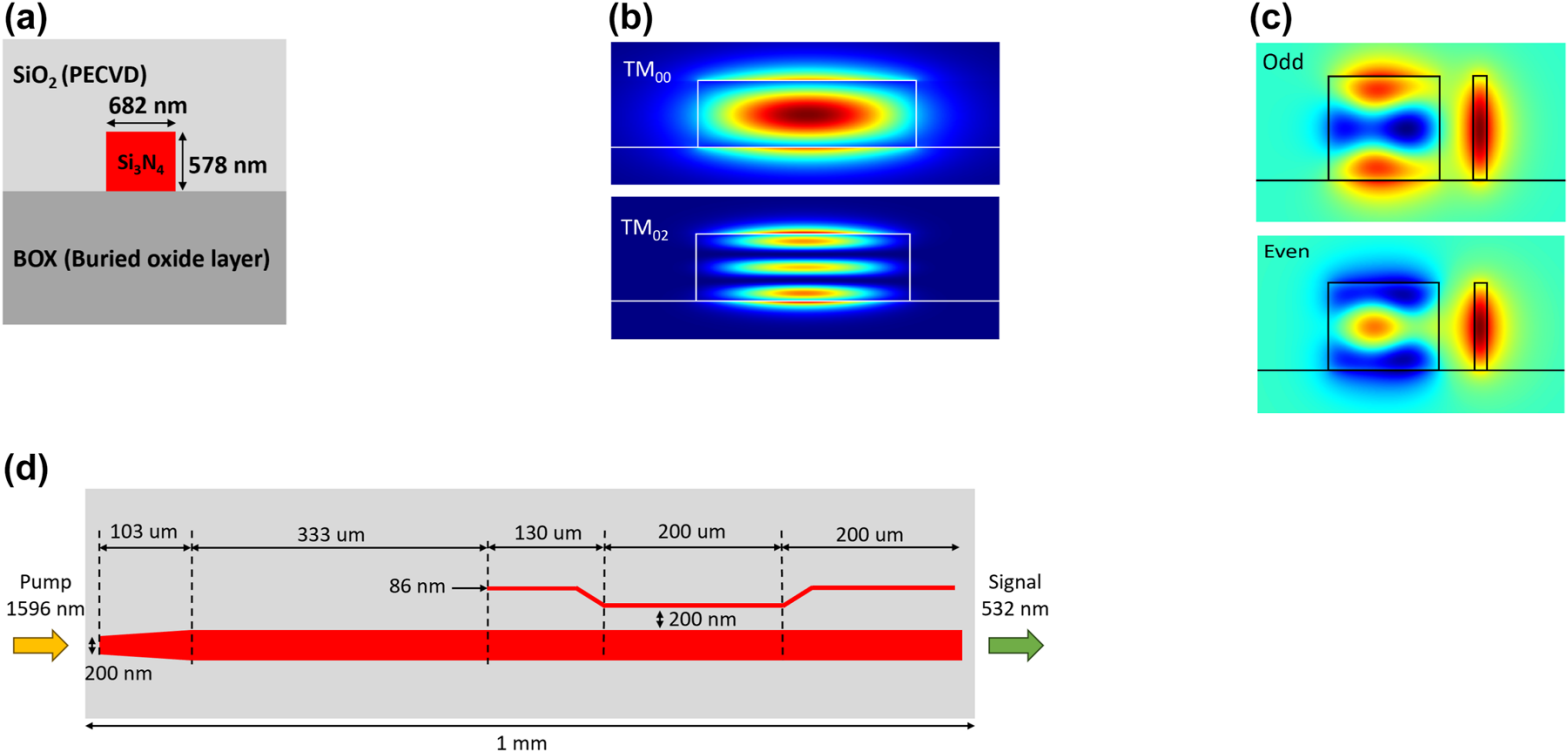
The schematic of the THG SiN waveguide designed to generate the third-harmonic at 532 nm from the fundamental at 1,596 nm. (a) The device cross-section, consisting of a bottom thermally grown fused silica layer, followed by the etched silicon nitride layer and clad by a layer of PECVD silicon dioxide. (b) Simulated spatial intensity profiles of TM_00_ mode at 1,596 nm and TM_02_ mode at 532 nm. (c) Simulated TM polarized electric field profile of the even and odd modes of the directional coupler. (d) The device layout includes an inverse taper to increase the mode size and match it with a focused free-space pump at 1,596 nm and a directional coupler at the output to convert the third-harmonic TM_02_ mode at 532 nm to a fundamental TM_00_ mode.

We designed and fabricated the silicon nitride waveguides for phase-matched THG on a fused silica substrate cladded by silicon dioxide, as shown in Figure 1(a). We calculated the effective refractive indices of guided modes using Lumerical MODE solutions based on the finite difference eigenmode (FDE) method [50]. The refractive indices of all the materials used in the simulation are based on a Sellmeier fit of the ellipsometry measurements of each material. We optimized the waveguide dimensions (height = 578 nm, width = 682 nm) to match the effective refractive indices (*n*_eff_ = 1.61713) of the TM_00_ mode at 1,596 nm and the TM_02_ mode at 532 nm. Their mode profiles are shown in Figure 1(b). From the simulated mode profiles and equations (1) and (3), we numerically calculated *A*_eff_ = 7.52 μm^2^ and *γ* = 377 W^−1^ km^−1^.

To convert the third-harmonic TM_02_ to a TM_00_ mode, we designed an 86-nm-wide directional coupler separated by 200 nm near the output end of the waveguide. We obtained the effective refractive indices of 1.617913 and 1.61598 for the even and odd modes (shown in Figure 1(c)), respectively. From equation (5), we calculated *L*_100 %_ = 138 μm, and established a coupling length of 200 μm to partially transmit the TM_02_ and verify its far-field spatial profile. We inverse-tapered the waveguide at the input end, gradually decreasing the waveguide width to 200 nm over a length of 103 μm to improve the IR mode coupling efficiency, as shown in Figure 1(b).

We grew a 5-μm-thick fused silica substrate on a silicon wafer in a thermal furnace and deposited a 578-nm-thick silicon nitride layer on top by liquid-phase chemical-vapor deposition (LPCVD). We annealed the wafers for 1 h under a nitrogen atmosphere at 650 °C to reduce the stress encountered during the deposition. We patterned the waveguides using e-beam lithography, varying the waveguide width in increments of 2 nm between 760 nm and 860 nm, with an 86-nm-wide directional coupler alongside all the waveguides. We deposited a 3-μm silicon dioxide layer on top of the fabricated waveguides using plasma-enhanced chemical-vapor deposition (PECVD).

## 4 Experiments

**Figure 2:**
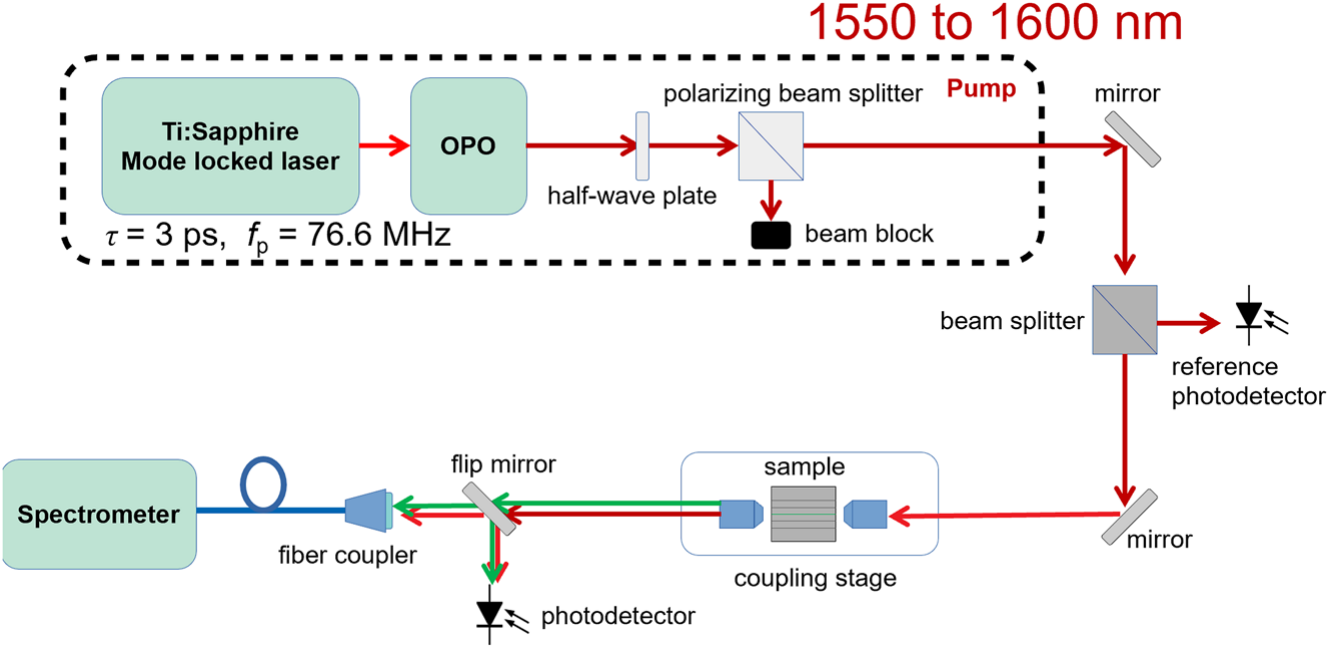
The experimental setup consisting of a pump source, coupling stage and spectrometer. 3-ps-wide (*τ*_p_) pump pulses are generated by an OPO, pumped by a Ti:Sapphire laser at a repetition rate of 76 MHz (*f*_p_). The average pump power is controlled by a half-wave plate and polarizing beam splitter. The light is coupled in and out of the waveguides by two 0.65 NA aspheric lenses. The collected signal spectrum is measured by an ultraviolet-visible spectrometer (Ocean Optics USB2000).

The experimental setup (shown in Figure 2) uses a titanium-sapphire-laser-pumped optical parametric oscillator (OPO) to generate mode-locked 3-ps-wide (*τ*_p_) pulses at a repetition rate of 76 MHz (*f*_p_). The central wavelength of the pulses is varied between 1,550 and 1,600 nm. A half-wave plate and polarizing beam splitter control the polarization and average power of the OPO output before it is coupled into the waveguide-on-chip by a 0.65 NA aspheric lens. The waveguide chip is mounted on a precision stage to optimize the pump’s coupling efficiency and switch between the waveguides on the chip. Light scattered from each waveguide is imaged on an overhead visible-range CMOS camera to visualize the light generated from the chip. The transmitted light is collected by an aspheric lens and coupled into a fiber-coupled ultraviolet-visible spectrometer (Ocean Optics USB2000). Two beam splitters pick off a fraction of light before it is coupled into the waveguide and fiber-coupled spectrometer to monitor the pump and signal power.

**Figure 3:**
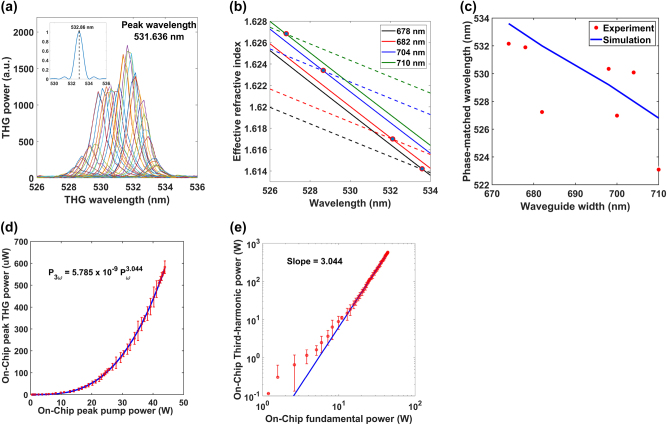
Experimental data of phase-matched THG spectrum and power scaling. (a) Aggregated spectra of signal output obtained by scanning the wavelength of the pump beam in increments of 0.5 nm. The inset shows the simulated sinc-squared dependence of the third-harmonic wavelength. (b) Effective refractive index plotted against the phase-matched third-harmonic wavelength, obtained by FDE simulations. The colors represent different waveguide widths at a fixed height of 578 nm, while dashed lines represent the fundamental mode at three times the third-harmonic wavelength and the solid lines represent the harmonic mode, respectively. (c) Waveguide-width-dependent phase-matched wavelengths with the solid line representing the simulated phase-matched wavelengths. The points correspond to experimental results in several different waveguides. (d) The signal peak power as a function of the pump peak power coupled into the waveguide of the width 678 nm. (e) Logarithmic plot of the data of part (d), linearly fit with a slope of 3.044.

The pump wavelength was tuned in steps of 0.5 nm from 1,550 to 1,600 nm. At each step, we measured the signal spectrum by the spectrometer and plotted the readings obtained from all the steps in Figure 3(a). The aggregated spectrum peaks at 531.63 nm, close to the simulated wavelength of 532.86 nm corresponding to a 678-nm-wide waveguide, and is compared to a sinc-squared wavelength dependence, as shown in the inset to Figure 3(a). The plot also shows a blue-shifted peak, which we attribute to the waveguide width taper along the interaction length at the inverse taper and directional coupler.

To measure the waveguide-width dependence of the phase-matched THG process, we sequentially excited several waveguides on the chip with different widths while tuning the pump beam’s center wavelength to match each waveguide’s phase-matched wavelength. We plot the phase-matched signal wavelength (measured by the peak of the aggregated spectrum of each waveguide) as a function of the waveguide width (Figure 3(c)) and compare the experimental measurements with the simulated wavelength dependence in Figure 3(b). The simulated dependence is obtained from the crossing we varied the average OPO power from 0–40 mW and measured the average power of the generated green wavelength signal on a power meter. The coupling-in efficiency of the IR light into the waveguides is estimated to be 30 %, based on the transmission measurements and the assumption that the scattering loss of the 1-mm-long waveguide is negligible. The out-coupling efficiency of the signal is 95 %, based on the calculation of Fresnel reflectance between the air-waveguide interface, where we assume the refractive index of the waveguide to be the effective refractive index (1.62) of the signal TM_02_ mode.

**Figure 4:**
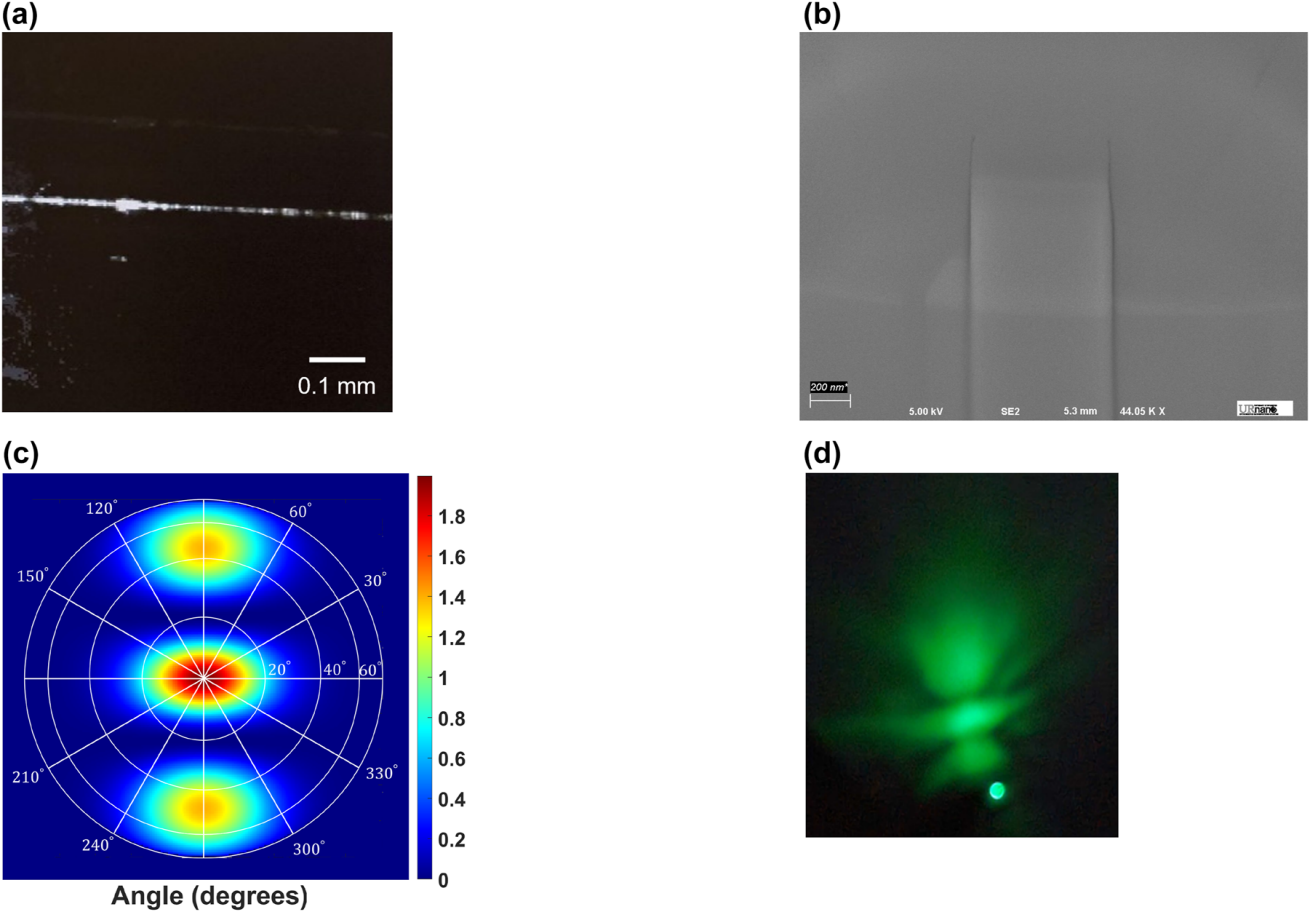
Images of sample characterized for modal phase matching. (a) The green light scattered from the waveguide is imaged onto an overhead visible-range CMOS camera. The light is seen to increase in brightness along the propagation length, as a result of quadratic increase in THG with the interaction length. (b) SEM image of the fabricated waveguide at the directional coupler. The image was taken after a portion of the cladding layer is removed by focused-ion beam milling. (c) The simulated far-field projection of the TM_02_ mode, in which harmonic light is designed to be generated. (d) The observed far-field profile of the third harmonic emitted from the waveguide.

We estimate the normalized internal THG efficiency by plotting the on-chip peak power of the emitted green light against the on-chip peak power of the IR pump light in Figure 3(d). The peak power (*P*_pk_) is calculated from the estimated average power (*P*_av_) in the waveguide, using *P*_pk_ = 0.88 *P*_av_/(*f*_p_ *τ*_p_). We confirm the cubic dependence of the signal peak power on the pump peak power, by calculating the slope (3.044) of a logarithmic plot (see Figure 3(e), displaying the signal versus pump powers). We estimate a peak-power-normalized THG efficiency of 5.78 × 10^−7^ %/W^2^ from an adjusted cubic fit of Figure 3(d) based on the slope of Figure 3(e). The THG efficiency was measured in the 578-nm-high and 678-nm-wide waveguide, as it generated the brightest signal spectrum. We observed an increase in the green-light emission along the propagation direction of the waveguide through the overhead camera Figure 4(a). We viewed the third-harmonic mode’s transverse profile on a screen close to the output facet of the chip, shown in Figure 4(c). The transverse profile matches the simulated far-field profile of a TM_02_ mode, as predicted by our FDE simulations (Figure 4(b)).

## 5 Discussion and outlook

We have demonstrated tunable phase-matched THG in silicon nitride waveguides in the visible wavelength range. Our experimental results match the trend predicted by the simulations. The far-field profile of the third-harmonic generated in the TM_02_ mode shows that the mode-engineered phase-matching of the THG in waveguides is possible. We have measured the spectrum of the generated third harmonic by scanning across different wavelengths. The plot deviates from the expected sinc-squared dependence on wavelength, peaking at the phase-matched wavelength. The deviations from the expected spectrum can be attributed to the width tapering and the presence of the directional coupler intended to convert the TM_02_ mode into a fundamental mode for a mode-matching with a potential application. We have shown that the generated harmonic power follows a cubic dependence on pump power without saturation up to a peak power of 40 W. We estimate an internal peak-power normalized conversion efficiency of 5.78 × 10^−7^ %/W^2^, accounting for the coupling losses under the assumption that the propagation losses are negligible, as expected from a 1-mm-long silicon nitride chip. The estimated efficiency is in close agreement with our calculated normalized conversion efficiency of 5.63 × 10^−7^ %/W^2^, given by

(6) $$\eta =\frac{\gamma^{2}L^{2}}{9},$$

where the effective nonlinearity of our waveguide is 337 W^−1^ km^−1^, based on the overlap calculations performed in FDE simulations. This value matches well with the effective nonlinearity of 345 W^−1^ km^−1^, estimated from the experimentally measured conversion efficiency.

To our knowledge, we have demonstrated, for the first time, phase-matched third-harmonic generation in a straight silicon nitride waveguide, by verifying the far-field profile of the third-harmonic mode. The extracted conversion efficiency is larger than that in other waveguide platforms, such as Si photonic crystals [17] and Si waveguides [18], but smaller than cascaded THG in PPLN waveguides [44] and high-Q resonators [14], [42], as shown in Table 1. However, high-Q resonators may not be suitable for ultrashort pulse conversion due to narrow resonance bandwidth, and cascaded THG requires careful design of the poling period.

**Table 1:** THG efficiency of different platforms.

Platform	Peak intensity (GW/cm^2^)	Efficiency (%)	Normalized efficiency (%/W^2^)
Si metasurface [14]	0.7	1.13 × 10^−3^	–
Si photonic crystal [17]	–	1 × 10^−5^	5 × 10^−8^
Si waveguide [18]	130.5	2.8 × 10^−3^	1.3 × 10^−7^
Si_3_N_4_/AlN resonator [42]	–	–	180
PPLN waveguide [44]	–	–	0.098
This work	4.26	4.39 × 10^−4^	5.78 × 10^−7^

The THG efficiency can be improved by increasing the length of our waveguides by coiling them within the e-beam write field. The waveguide length (*L*_opt_) is limited by the estimated loss at the pump (*α*_p_) and signal (*α*_s_) wavelengths, given by [46]

(7) $$L_{\text{opt}}=\left(\frac{1}{\alpha_{\text{p}}-3\alpha_{\text{s}}}\right)\ln\left(\frac{\alpha_{\text{p}}}{3\alpha_{\text{s}}}\right).$$

We estimate an optimal length of 4 cm, assuming the losses of 0.1 dB/cm and 3 dB/cm [51] at the pump and signal wavelengths, respectively. The estimated conversion efficiency can be further enhanced by fabricating waveguides on low-stress Si-rich nitride platforms [37], which possess a large nonlinear susceptibility of 1.4 × 10^−18^ m^2^/W while remaining transparent at 532 nm. The conversion efficiency in our device is further limited by scattering losses in our directional couplers, which collapsed during the fabrication due to the large aspect ratio between the width (86 nm) and height (578 nm) of the coupler. We propose modifying the design by fabricating directional couplers above the THG waveguide with the same width as the waveguide and a small height. Changing the design, as stated above, can improve the normalized conversion efficiency up to 3 × 10^−2^ %/W^2^. THG on our platform is suitable for integrated metrology, sensing and nonlinear microscopy. Phase-matched silicon nitride waveguides can be combined with frequency combs for on-chip *f* − 3*f* self-referencing [5]. The waveguides can be integrated into endoscopes for increased imaging depth using third-harmonic microscopy [2]. Our phase-matching scheme is also suitable for generating GHz-entangled photons on a scalable CMOS platform [52]. The sensitivity of the phase-matched wavelength to material parameters could also be used to sense the presence of chemicals that perturb the initial phase-matched wavelength of the system.
